# Demonstration of Shot-noise-limited Swept Source OCT Without Balanced Detection

**DOI:** 10.1038/s41598-017-01339-6

**Published:** 2017-04-26

**Authors:** Vala Fathipour, Tilman Schmoll, Alireza Bonakdar, Skylar Wheaton, Hooman Mohseni

**Affiliations:** 1 Bio-Inspired Sensors and Optoelectronics Laboratory, Northwestern University, 2145 Sheridan Rd, Evanston, IL 60208 USA; 2 ZEISS, Medical Technology Business Group, Advanced Development, 5160 Hacienda Drive, Dublin, CA 94568 USA

## Abstract

Optical coherence tomography (OCT) has been utilized in a rapidly growing number of clinical and scientific applications. In particular, swept source OCT (SS-OCT) has attracted many attentions due to its excellent performance. So far however, the limitations of existing photon detectors have prevented achieving shot-noise-limited sensitivity without using balanced-detection scheme in SS-OCT, even when superconducting single-photon detectors were used. Unfortunately, balanced-detection increases OCT system size and cost, as it requires many additional components to boost the laser power and maintain near ideal balanced performance across the whole optical bandwidth. Here we show for the first time that a photon detector is capable of achieving shot noise limited performance without using the balanced-detection technique in SS-OCT. We built a system using a so-called electron-injection photodetector, with a cutoff-wavelength of 1700 nm. Our system achieves a shot-noise-limited sensitivity of about −105 dB at a reference laser power of ~350 nW, which is more than 30 times lower laser power compared with the best-reported results. The high sensitivity of the electron-injection detector allows utilization of micron-scale tunable laser sources (e.g. VCSEL) and eliminates the need for fiber amplifiers and highly precise couplers, which are an essential part of the conventional SS-OCT systems.

## Introduction

Optical coherence tomography (OCT) is a tomographic imaging technique that has revolutionized biomedicine since its invention in 1991^1–3^. It provides images with micron-scale resolution, and up to few millimeters of tissue depth. OCT has the outstanding property of decoupling depth resolution from transverse resolution. Furthermore, the interferometry technique allows imaging weakly scattering structures with a high sensitivity (about −100 dB) and dynamic range.

Despite its short history, OCT’s great imaging ability has resulted in rapid growth and significant progress in this field. Over the last decade, optical coherence tomography has become the workhorse of various fields including biology^4^, medicine^5^, manufacturing inspection^6^, and physical sciences. In particular, OCT has made a profound impact as a diagnostic tool in ophthalmology^7, 8^, cardiology^9^, dermatology^10^, dentistry^11, 12^, and ‘*in-situ*’ optical biopsy^13^.

One important figure of merit in an OCT system is the signal-to-noise-ratio (SNR), where the signal is proportional to the optical power from the sample and the noise is defined as the variance of the background. The largest possible SNR for a given sample power is obtained under the shot-noise-limited regime, where the overall system noise is dominated by the photon shot noise coming back from the sample arm. Unfortunately, the electrical noise of the photon detectors, and the electronics attached to them, can significantly reduce the SNR below this limit - particularly, if the light source cannot provide sufficient power to the reference arm. For example, standard photodiodes used in the traditional OCT systems at 1300 nm, need a reference power in the order of few miliwatts to approach close to shot-noise-limited sensitivities^14, 15^. To reduce the noise level, Mohan *et al*., used superconducting single-photon detectors (SSPDs) in a Michelson interferometer and demonstrated nearly shot-noise-limited operation with a single SSPD at about 10 nW reference optical power. However, SSPDs are expensive and bulky, as they require cryogenic cooling down to 1.8 K. Furthermore, SSPDs have a limited bandwidth, which is not enough for real-time acquisition of large volumetric datasets^16^.

Currently, the fastest OCT imaging systems utilize swept source lasers (SS-OCT)^7, 17–20^. SS-OCT enables reduced sensitivity to patient motion, and allows deeper imaging^7^ compared with other OCT modalities. Since the signal power produced by the photodetector in an OCT system is proportional to the intensity of the reference beam, a high reference power is typically needed to amplify the signal above the detector noise. This requirement is even more significant in SS-OCT due to the higher noise of fast photodetectors needed in a high-speed system. Since the intensity fluctuation noise power is also proportional to the square of reference beam power, balance detection is needed to cancel out the resulting intensity-fluctuation noise term. However, balanced detection requires a complex interferometer arrangement with expensive components that maintain their balanced performance across a wide spectral range, along with careful adjustment of the balanced receiver to subtract out the large amount of intensity noise. In practice, residual non-canceled output of excess photon noise of 20–40% has often been reported in the literature^17, 21, 22^. As such, achieving shot-noise-limited operation at low source powers has been long sought for.

Here, we utilize a new photon detector to demonstrate shot-noise-limited sensitivity in SS-OCT system without balanced detection and at room temperature for the first time. Shot-noise-limited performance is achieved at what we believe is the lowest reference power level reported in SS-OCT systems.

The detector has a very large internal amplification^23, 24^ that enhances the signal well above the receiver electronic noise, even for a reference beam power of ~350 nW. We compare our results with those of a commercial p-i-n detector that was measured simultaneously in the same setup. Theoretical calculations show excellent agreement with our experimental results.

Our approach, can immediately address the demand for a portable OCT system, which has been the subject of much recent scientific attention^25–28^. It eliminates the concomitant complexity and size of typical OCT systems. In addition to eliminating many passive components required for balanced detection scheme, the low power requirement of our approach would allow replacing the typical large-footprint high-power OCT laser sources with an electrically pumped tunable VCSEL^29, 30^ to radically reduce the size and cost of OCT systems.

## Experimental

### Results and Discussions

We evaluated the performance of an electron-injection (EI) detector and then utilized it in a swept source OCT system. EI detectors were introduced in 2007^31^. They have an internal amplification mechanism that is not based on avalanche. Therefore, they require very low operating voltages^32^ and do not add any extra noise during amplification^33^. EI detectors utilize similar device micro-processing and material system as the conventional p-i-n photodiodes (see Methods). The EI detectors used in this study have a cutoff wavelength of 1700 nm operate at room temperature, and hence could be used in majority of the existing near-infrared and short-infrared OCT systems. EI detectors with shorter cutoff wavelengths have even better noise performance.

#### Experimentally Measured Noise Equivalent Power of EI Photodetectors

**Figure 1:**
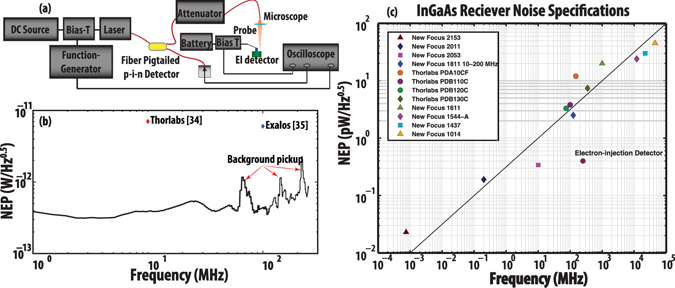
(**a**) Small-signal homodyne setup for measurement of electron-injection detector responsivity. The spectral shot noise current of the EI detector was also measured in the same setup using a spectrum analyzer. Red lines show optical connections and black lines show electrical connections. (**b**) The extracted noise equivalent power of the EI detector versus frequency. The noise equivalent power of the EI detector is as low as ~300 fW/√Hz at room temperature. The high-frequency peaks show the background pick up by the un-shielded probe. The noise equivalent power of two commercially available balanced detectors with a similar cutoff wavelength is also marked. NEP of the BDs is more than an order of magnitude higher than the EI detector. This suggests that to obtain same sensitivity, more than 2 orders of magnitude higher reference arm power should be used for the balanced detectors^34, 35^. (**c**) A comparison of the electron-injection detector noise equivalent power with a number of commercial detectors.

Outside the shot-noise-limit, improving the noise equivalent power (NEP) of the photon detector has a significant impact on the OCT system performance. This is because the system sensitivity improves with the square of the detector NEP. Under shot-noise-limited condition, reducing NEP leads to a reduction of the required reference power. Therefore, in order to evaluate the merits of using electron-injection detectors in an SS-OCT system, we measured the noise equivalent power of the EI detector and compared it to the state-of-the art balanced photodetectors at frequencies relevant to high-speed SS-OCT systems. To obtain NEP we used the well-known formula $\,NEP={I}_{n}/ {\mathcal R}$. In this equation, ${\mathcal R}$ is the detector responsivity, and *I*_*n*_ is the spectral noise of the detector. We used a small-signal homodyne measurement approach shown in Fig. 1(a) to measure detector responsivity from 100 KHz to 250 MHz. In this setup, we used a DC voltage source to provide the bias voltage for our tunable laser, and a function generator to provide the small-signal voltage swing. The monitoring p-i-n detector ensures delivery of constant optical power to the EI detector at all frequencies. The spectral noise of the detector was further measured in the same setup using a spectrum analyzer. The extracted noise equivalent power of the EI detector is shown in Fig. 1(b). The NEP of the EI detector is as low as 300 fW/√Hz at room temperature. Since, the EI detector is not currently a packaged device, the detector was probed inside a microscope setup. The high-frequency peaks in Fig. 1(b) are due to the background radio signals picked up by the un-shielded probe. For comparison, the reported noise equivalent power of a few commercially available balanced detectors with a similar cutoff wavelength is shown in Fig. 1(b) ^34, 35^.

It is worth noting that since the conception of OCT, the detectors used in these systems have not seen considerable improvement. In fact, the noise equivalent powers of balanced detectors with bandwidths of higher than 100 MHz have been nearly constant (at about ~3–10 pW/√Hz), over the past 20 years^17, 18, 34–36^. A comparison of the electron-injection detector noise equivalent power with a number of commercial detectors is illustrated in Fig. 1(c). The better NEP of the EI detectors suggests that for the same reference arm power, the minimum detectable OCT signal using the balanced detectors would be more than two orders of magnitude worse than the EI detector. Equivalently, to obtain same sensitivity, more than 2 orders of magnitude higher reference arm power is needed for the balanced detectors approach.

#### SS-OCT Experimental Setup

**Figure 2:**
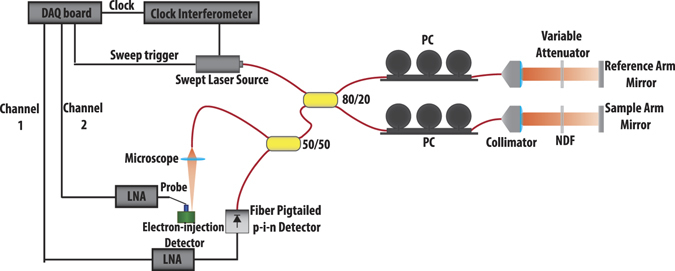
Schematic diagram of our SS-OCT measurement setup for comparing the electron-injection detector with the commercial p-i-n detector: Swept laser source has a central wavelength of 1060 nm, with 110 nm tuning range and 100 kHz scan rate. In order to be able to properly compare the SNR performance of our detector and a commercial fiber pigtailed p-i-n detector, a second directional coupler (50/50) was used to simultaneously send the interference signal to both detectors. Light was coupled to the electron-injection detector via a microscope set up, which added optical loss. NDF: Natural Density Filter, PC: Polarization Controller, LNA: Low Noise Amplifier, DAQ: data acquisition board.

We evaluated the performance of EI detectors in an actual SS-OCT setup. Figure 2 shows the schematic diagram of this system. The output of a swept laser source is split via a directional coupler (splitting ratio 80/20), into the reference arm and the sample arm, which illuminates and receives the light reflected from the sample. We use a single reflector (mirror) and a neutral density filter (NDF) to mimic the sample. The reference power is varied by means of a variable neutral density filter placed in front of the reference arm reflector. The swept source laser has a sweep rate of 100 KHz and a center wavelength of 1060 nm. The theoretical limit for the free space axial resolution, and ranging depth are 4.9 μm and 3.7 mm respectively in our setup.

The interference between the reference arm and sample arm signals is detected with the photodetector. In order to properly compare the SNR performance of the electron-injection detector and a commercial p-i-n detector, we used a second directional coupler (splitting ratio 50/50) to simultaneously send the interference signal to both devices. Light was coupled to the electron-injection detector via a microscope set up with ~1.6 dB optical loss. The p-i-n detector was fiber pigtailed, shielded in a metallic box, and had a coaxial cable connection. Polarization controllers (PCs) were used to account for the polarization mismatch and improved the shape and the peak height of the interference envelope. Outputs of the p-i-n and the EI detectors were amplified simultaneously using voltage amplifiers. Electronic band pass filtering was implemented to improve the SNR. The signals were then digitized using a data acquisition board (DAQ). In our setup, the input referred noise of the amplifiers, dominated the quantization noise of the DAQ.

The key parameters defining the system performance (see “Methods” section, equation (6), for details) were experimentally measured and presented in Table 1 for both EI and p-i-n detectors.

**Table 1:** Key parameters defining the system SNR performance.

Parameter	Electron-Injection Detector	p-i-n Detector
P_s_(pW)	160	320
${i}_{amp}(pA/\sqrt{Hz})$	10	7
η_ext_	78%	60%
η_int_	100%	100%
G(at 35 MHz)	70	1
F	1	1
P_RIN_(dBc/Hz)	−130.6	
BW(KHz)	100	
P_0_ (mW)	0.89	

#### Experimentally Measured Sensitivity in SS-OCT Setup

**Figure 3:**
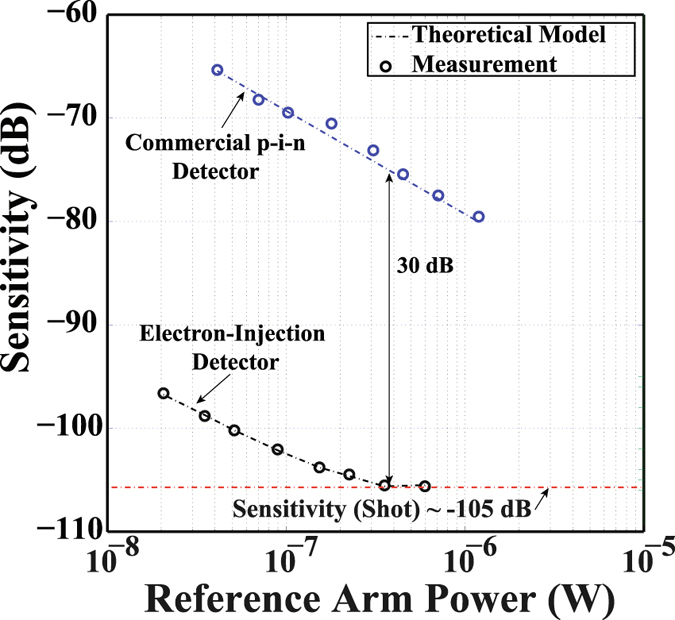
Comparison of the measured and the theoretically calculated sensitivity as a function of the reference arm power for two single-ended detection schemes (electron-injection detector and the commercial p-i-n detector). The symbols are experimental data and curves are theoretically calculated. Electron-injection detector yields ~30 dB higher sensitivity compared with the commercial p-i-n detector. Sample power at the electron-injection detector was ~160 pW. The gain in our device amplifies shot noise and allows it to surpass amplifier noise at a factor of *G* ^2^ lower reference power level compared to the p-i-n diode. EI reaches shot noise limited sensitivity of ~−105 dB at reference power level of 350 nW. This is, to best of our knowledge, the lowest reference power ever achieved for a shot-noise-limited operation in swept laser source OCT. Furthermore, this is first report of shot-noise-limited performance, without the use of balanced detection in swept source OCT.

Figure 3 shows the experimental results for sensitivities of the EI and the p-i-n detector, measured simultaneously as a function of the reference arm power at the detectors. To determine the optimum reference arm power, signal-to-noise-ratio of the detector was obtained from the Discrete Fourier Transform (DFT) of the sampled detector signals as a function of the reference arm power. The reference arm power at EI detector, was varied from ~20 nW to ~600 nW using the variable neutral density filter placed in front of the reference mirror. The total attenuation level of the sample arm was ~67.5 dB and the power returning from the sample at the electron-injection detector was 160 pW. The experiment was performed at a beating frequency of 35 MHz.

As the reference arm power is increased, SNR increases until it reaches its maximum, dictated by the sample power returning to detector (*P*_*s*_). The gain in our device amplifies shot noise and allows it to surpass the amplifier noise at a factor of *G* ^2^ times lower reference power compared to the p-i-n detector.

Utilizing the measured detector parameters provided in Table 1, in equation (4) of the “Methods” section, one can conclude that EI detector shot noise would surpass amplifier noise (also provided in Table 1), at a reference power of 350 nW. This is confirmed experimentally in our SS-OCT system. As shown by the markers in Fig. 3, the measured sensitivity of the EI detector reaches its shot-noise-limit of ~−105 dB (indicated by the red dotted line, and calculated from equation (7) in the “Methods” section) at a reference power level of ~350 nW. To best of our knowledge, this is the lowest reference power ever achieved for a shot-noise-limited operation in SS-OCT systems at room temperature. The typical optimum reference power level for a balanced detection system is in the few mW range^14, 15, 37^ with the lowest reported value of ~15 μW^17^. Furthermore, this is the first report of shot-noise-limited performance, without the use of balanced detection in an SS-OCT setup.

As shown in this figure, an improvement in sensitivity of ~30 dB is obtained by the electron-injection detector compared with the commercial p-i-n detector. The theoretically calculated sensitivity curves, obtained from the measured data presented in Table 1, and using equation (6) in the “Methods” section, as well as the attenuation in sample arm, are indicated by the dotted lines in Fig. 3.

**Figure 4:**
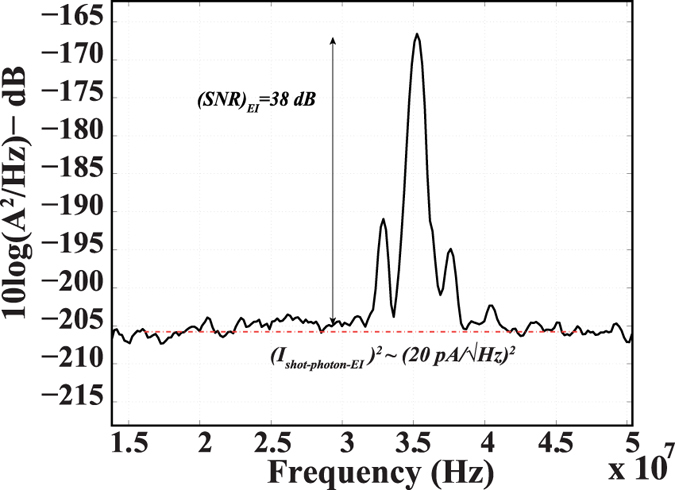
Measured A-line profile at a reference arm power of 350 nW at the electron-injection detector. The sample arm power at the electron-injection detector was 160 pW. The theoretical shot noise power at the electron-injection detector due to the statistical nature of incoming photons is marked via the red dotted line. The experimentally measured shot noise power lies at this ideal value.

Figure 4 depicts the A-line profile at a reference arm power of 350 nW obtained from the EI detector. The measured SNR of ~38 dB is in good agreement with the theory for shot-noise-limited SNR (equation (6) in the “Methods” section). From the measured SNR, and the attenuation in sample arm, sensitivity of system under the shot-noise-limit is confirmed to be about −105 dB﻿.﻿ Furthermore, the experimentally measured noise data lie around the red dotted line (equation (4) in the “Methods” section), which shows the theoretically calculated noise power for our detector operating under shot-noise-limited regime.

From the measured sensitivity, one can conclude that EI detector could respond to powers coming from sample arm as low as 25 fW at 35 MHz bandwidth (P_0_=0.89 mW). In our experimental setup, measurements at sample powers lower than 160 pW were limited by the non-ideal directivity of the coupler.

From the measured shot noise in Fig. 4 and the measured EI responsivity from Table 1, the NEP of the detector can be confirmed to be about 300 fW/√Hz. This is similar to the value obtained from the homodyne approach.

The side lobes in the interference fringe envelope in Fig. 4, which were also present in the p-i-n detector spectra, are possibly caused by the non-ideal Gaussian spectral shape of the source. Furthermore, group velocity dispersion and polarization mismatch between the reference and the sample arms are factors that strongly affect the interference envelope^38^.

**Figure 5:**
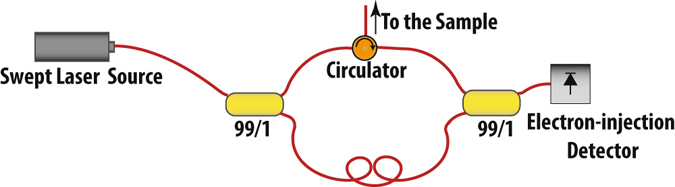
Schematic of a compact OCT system utilizing the electron-injection detector.

We would like to emphasis that the above OCT setup was designed to allow simultaneous evaluation of EI and p-i-n photodetectors. Therefore, it uses a high power swept source laser and an additional coupler. However, our results suggest that a compact OCT system with fewer components and significantly lower laser power can be realized. A schematic diagram of such system is shown in Fig. 5. Current wavelength-swept VCSELs can provide up to few milliwatts of optical power^39, 40^. However, booster optical amplifiers (BOAs) are an integral part of almost any SS-OCT system today^41–43^, since the VCSEL output power needs to be amplified to higher than 20 mW^42^. For applications that require few milliwatts sent to the sample arm, such as in ophthalmology, a system based on the EI detector can utilize a single VCSEL where almost all of the VCSEL power could be directed to the sample arm using a 99/1 coupler. Our models predict that sensitivities of better than −100 dB could be obtained, using a single micro-scale electrically pumped VCSEL^29, 30^ and eliminating the need for optical power amplification. Such a system has also a smaller footprint, lower cost and complexity, as it would eliminate the need for utilizing two channels that require near-perfect matching with regard to the optical signals on each detector in different polarization, gain, and noise across the whole optical bandwidth^17, 21, 22^.

For applications that require more than few milliwatts of optical power on the sample arm, the reduced laser power might not necessarily be the advantage. In such applications however, utilization of the EI detector would still make the system simpler, as it would eliminate the need for having two perfectly matched channels.

To conclude, progress towards a compact and low-cost swept laser source OCT system has long been limited by the lack of high performance detector technologies. Electron-injection detectors reduce the contribution of post-detection circuitry noise by several orders of magnitude so that it becomes irrelevant even at low optical powers. EI detectors have a cutoff wavelength of 1700 nm; operate at room temperature and at low bias voltages. They provide a noise-free stable internal amplification mechanism with unity excess noise. Using these detectors, we experimentally demonstrated shot-noise-limited sensitivity in SS-OCT system without balanced detection for the first time. This was achieved at ~350 nW of optical reference power, which is believed to be the lowest power level reported in an SS-OCT operating at the shot-noise-limit. These experimental results show an excellent agreement with the theoretical calculations and suggest that EI detector could eliminate the concomitant cost and complexity of current SS-OCT systems. This detection approach can be an enabling technology for portable OCT systems, allowing use of a micron-scale tunable laser source (e.g. VCSEL) and a micron-scale detector.

## Methods

### Electron-injection Detector Layer Structure and Fabrication Procedure

The current device is composed of 1000 nm of n^−^ doped In_0.53_Ga_0.47_As absorber, 50 nm of p^+^ doped GaAs_0.52_Sb_0.48_ trapping layer, 50 nm of undoped In_0.52_Al_0.48_As etch-stop layer, 500 nm of n^+^ doped InP injector, and 50 nm n^+^ doped In_0.53_Ga_0.47_As cap layer. Layers are grown by metal organic chemical vapor deposition on 2-inch InP substrates^24^.

**Figure 6:**
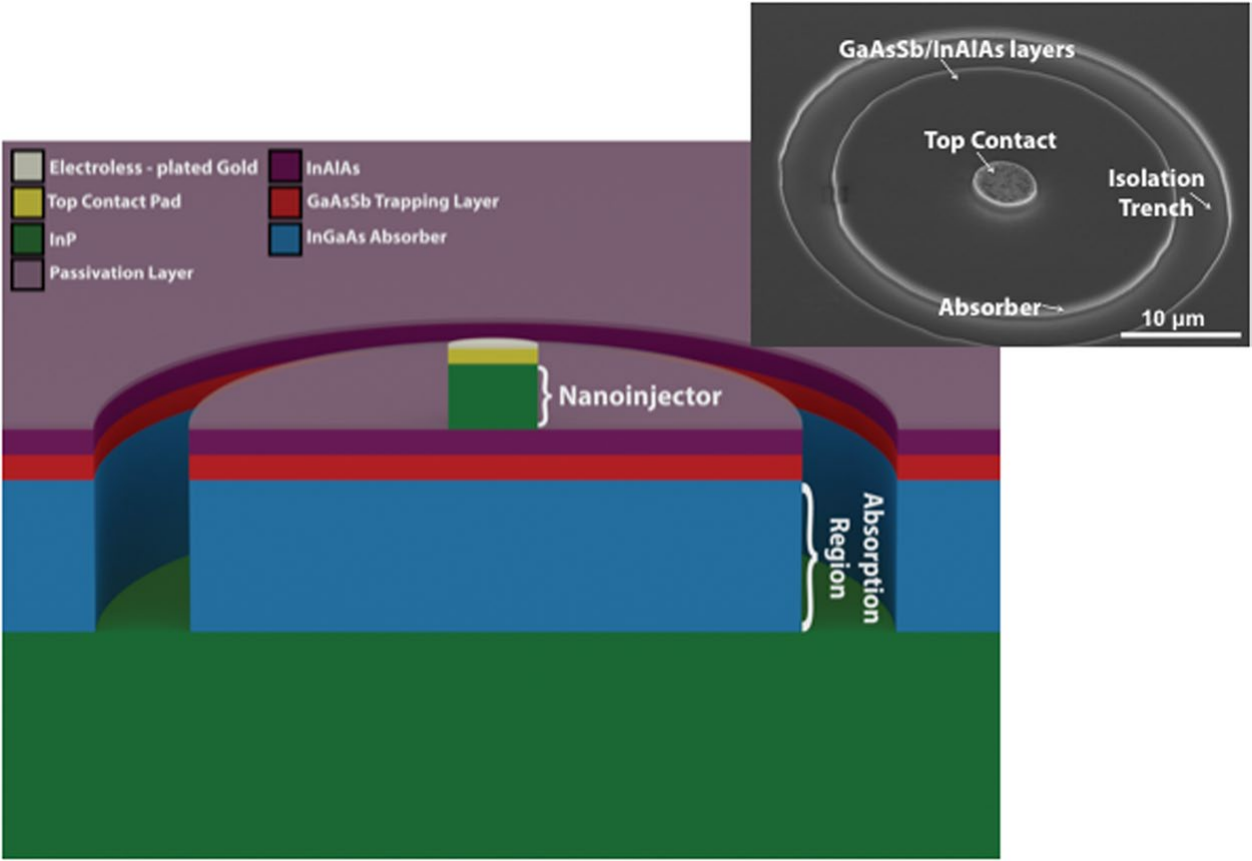
(**a**) Schematic diagram of passivated electron-injection detector with 5 μm injector diameter and 30 μm absorber diameter. The scanning electron microscope image is taken before passivation and electroless plating.

Devices are fabricated by patterning the wafers with e-beam lithography to form the contact metals. Conventional metallization with an E-beam evaporator is used to lift off multi-layer metal contacts, which act as hard mask for reactive ion etching with CH4/H2 chemistry to form the injector pillars. Wet etching of InAlAs and GaAsSb followed by a CH4/H2 dry etching of InGaAs is then used to define the absorber volume. Finally, the detectors are passivated. For robust direct probing of detectors, electroless plating is used to convert the top Nickel contact to gold. Figure 6 shows schematic of the electron-injection detector with 5 μm injector diameter and 30 μm absorber diameter that was used in this experiment. Scanning electron microscope is shown in the inset of Fig. 6, before passivation. All results reported here are based on devices operating at room temperature.

### Details of NEP Measurement Setup Equipment

A 1550 nm DFB, Butterfly laser from JDSU was biased through a DC source. A function generator (Gigatronics 6061A) provided the small signal swing. A bias-T (ZFBT-6GW from Mini-Circuits) was utilized to separate DC bias from the small signal AC signal. A 1550 nm 2 × 2 coupler (from Thorlabs) was utilized to send the modulated optical signal to the EI detector and a p-i-n detector simultaneously. We used a digital attenuator with 0.01 dB accuracy (NIST calibrated) to be able to vary the power on the EI detector. Frequency response was recorded using a real-time scope with a 2.5 GHz bandwidth (Agilent Technologies MSO9254A). For the noise measurement, we utilized a spectrum analyzer from Anritsu (Anritsu MS2717A).

### Details of SS-OCT Setup Equipment

Our swept laser source (from Axsun Technologies) was followed by (AC Photonic, Inc) optical isolator. The light output was then launched into a 2 × 2 coupler (from Thorlabs). The sample and reference arms were arranged with fiber collimators and mirrors. The mirrors and collimators were mounted on (KM100-Mount from Thorlabs) kinematic stages to allow coupling back to fibers. To alter the polarization of the transmitted light in the single mode fibers, two 3-Paddle Polarization Controllers (from Thorlabs) were utilized on sample and reference arm fibers. The interference signal from sample and reference arms was incident on the commercial p-i-n detector as well as the electron-injection detector. Outputs of the p-i-n and the EI detectors were amplified simultaneously using (DUPVA 1–70) low-noise voltage amplifiers (from Femto). Electronic bandpass filtering was implemented using (250 MHz low pass filter from Mini-Circuits and 2 MHz high pass filter from TTE Filters), to improve the SNR. Data was digitized using a data acquisition board (ATS9350, Alazar Technologies, Inc.) with a 12-bit resolution and a sampling rate of 500 mega samples per second. For each channel, a total of 1376 data points were obtained on each wavelength sweep. Background spectrum of the each channel was also obtained using 1376 data points. After subtraction of background spectrum, the resulting spectrum was reshaped by a Hann window, and inverse discrete Fourier transformed to give a single depth profile of the sample.

### Theoretical Analysis

As illustrated in Fig. 2, the interference signal that results from the mixing of the reference and the sample arm beams, which carries the information of interest, is incident on the electron-injection detector. To obtain a relation for the electron-injection detector’s current, we assume that the sample mirror is located at the axial coordinate z = z_0_ and z = 0 corresponds to zero optical path length difference between the two interferometric arms. We express the current of a detector with internal amplification *G*, which is detecting the interference signal as (1):

1 $${i}_{detector}(t)=\frac{\eta q}{h\nu }.\,[G.{P}_{r}+G.{P}_{0}.r{({z}_{0})}^{2}+2.G.\sqrt{{P}_{0}.r{({z}_{0})}^{2}.{P}_{r}}.\,\cos (2k(t){z}_{0}+\phi (\,{z}_{0}))]$$

As indicated in (1), the internal amplification, *G*, effectively boosts the weak signal reflected from the sample, even if the reference signal is not strong. In (1), *r*(*z*_0_)^2^ denotes sample arm reflectivity, *φ*(*z*_0_) is the interferometric phase shift associated with the detector signal, $k(t)=\frac{2\pi }{\lambda (t)}$ is the wave number, which is varied in time monotonically by tuning of the laser, *P*_*r*_ is the time average optical power over one tuning cycle from the reference arm at the detector, *P*_0_ is the time average optical power over one tuning cycle illuminating to the sample arm. Term $\frac{\eta q}{h\nu }$ denotes current to power conversion factor, where *η* is the quantum efficiency, *hν* is the photon energy and *q* is the electron charge. The first and second terms in (1) contribute to the non-interference background and the third term represents the interferometric signal. We express the detector’s signal current, *i*_*s*_(t), and noise power, ${{i}_{n}}^{2}$(t) as (2) and (3) respectively:

2 $${i}_{s}({\rm{t}})=\,\frac{\eta q}{h\nu }.G\mathrm{.2.}\sqrt{({P}_{s}.{P}_{r})}.\,\cos (2k(t){z}_{0})$$

3 $$\,\langle {{i}_{n}}^{2}({\rm{t}})\rangle ={{i}_{shot-photon}}^{2}+{{i}_{RIN}}^{2}+{{i}_{amp}}^{2}$$

In (2), *P*_*s*_ = *P*_0_. *r* ^2^ denotes the optical power reflected from the sample at the detector with the condition *P*_*s*_ ≪ *P*_*r*_. In (3), brackets < > denote a time average. The total noise current of detector is composed of the noise current due to statistical nature of incoming photons (*i*_*shot*−*photon*_) expressed as (4), intensity fluctuation noise term (*i*_*RIN*_) expressed as (5), and the electrical noise from the post-detection circuitry, which is dominant at low reference power levels (*i*_*amp*_).

4 $${i}_{shot-photon}={(2.q.\frac{\eta q}{h\nu }.{G}^{2}.({P}_{r}+{P}_{s}).BW.F)}^{1/2}$$

5 $${i}_{RIN}={({P}_{RIN}.{(\frac{\eta q}{h\nu })}^{2}.{G}^{2}.{({P}_{r}+{P}_{s})}^{2}.BW.F)}^{1/2}$$

Where, *P*_*RIN*_ is the relative intensity noise given in unit of *Hz* ^*−1*^, *BW* is the detection bandwidth, and *F* is the excess noise factor, which is a measure of deviation from the predicted shot noise level. The signal-to-noise ratio of the electron-injection detector is thus expressed as (6):

6 $$\begin{array}{rcl}{(SNR)}_{EI} & = & \frac{\langle {{i}_{s}}^{2}({\rm{t}})\rangle }{\langle {{i}_{n}}^{2}({\rm{t}})\rangle }\\  & = & \frac{2.{(\frac{\eta q}{h\nu })}^{2}.({P}_{s}.{P}_{r}).{G}^{2}}{[{{i}_{amp}}^{2}+2q.\frac{\eta q}{h\nu }.G{}^{2}.({P}_{r}+{P}_{s}).F+{P}_{RIN}.{(\frac{\eta q}{h\nu })}^{2}.G{}^{2}.F{({P}_{r}+{P}_{s})}^{2}].BW}\end{array}$$

The sensitivity is defined as the reflectivity that produces signal power equal to the noise power, and under shot-noise limited operation, is described by equation (7):

7 $${(Sensitivity)}_{EI}[dB]=-10\,\mathrm{log}\,\frac{\eta {P}_{0}}{h\nu .BW}$$
